# S‐Block Metal Mg‐Mediated Co─N─C as Efficient Oxygen Electrocatalyst for Durable and Temperature‐Adapted Zn–Air Batteries

**DOI:** 10.1002/advs.202403865

**Published:** 2024-07-04

**Authors:** Henan Wang, Xinxin Niu, Wenxian Liu, Ruilian Yin, Jiale Dai, Wei Guo, Chao Kong, Lu Ma, Xia Ding, Fangfang Wu, Wenhui Shi, Tianqi Deng, Xiehong Cao

**Affiliations:** ^1^ College of Materials Science and Engineering, Pinghu Institute of Advanced Materials Zhejiang University of Technology Hangzhou 310014 P. R. China; ^2^ College of Chemical Engineering Zhejiang University of Technology Hangzhou 310014 P. R. China; ^3^ Center for Membrane and Water Science and Technology College of Chemical Engineering Zhejiang University of Technology Hangzhou 310014 P. R. China; ^4^ State Key Laboratory of Silicon and Advanced Semiconductor Materials & School of Materials Science and Engineering Zhejiang University Hangzhou 310027 P. R. China; ^5^ Institute of Advanced Semiconductors & Zhejiang Provincial Key Laboratory of Power Semiconductor Materials and Devices Hangzhou Global Scientific and Technological Innovation Center Zhejiang University Hangzhou 311215 P. R. China

**Keywords:** metal─N─C, oxygen reduction reaction, *s*‐block, temperature‐adapted, Zn–air batteries

## Abstract

In the quest to enhance Zn–air batteries (ZABs) for operating across a wide spectrum of temperatures, synthesizing robust oxygen electrocatalysts is paramount. Conventional strategies focusing on orbital hybridization of *d*–*d* and *p*‐*d* aim to moderate the excessive interaction between the *d*‐band of the transition metal active site and oxygen intermediate, yet often yield suboptimal performance. Herein, an innovative *s*‐block metal modulation is reported to refine the electronic structure and catalytic behavior of Co─NC catalysts. Employing density functional theory (DFT) calculations, it is revealed that incorporating Mg markedly depresses the *d*‐band center of Co sites, thereby fine‐tuning the adsorption energy of the oxygen reduction reaction (ORR) intermediate. Consequently, the Mg‐modified Co─NC catalyst (MgCo─NC) unveils remarkable intrinsic ORR activity with a significantly reduced activation energy (*E*a) of 10.0 kJ mol^−1^, outstripping the performance of both Co─NC (17.6 kJ mol^−1^), benchmark Pt/C (15.9 kJ mol^−1^), and many recent reports. Moreover, ZABs outfitted with the finely tuned Mg_0.1_Co_0.9_─NC realize a formidable power density of 157.0 mW cm^−2^, paired with an extremely long cycle life of 1700 h, and an exceptionally minimal voltage gap decay rate of 0.006 mV h^−1^. Further, the Mg_0.1_Co_0.9_─NC‐based flexible ZAB presents a mere 2% specific capacity degradation when the temperature fluctuates from 25 to −20 °C, underscoring its robustness and suitability for practical deployment in diverse environmental conditions.

## Graphical abstract

A delocalized *sp* orbitals modulation strategy is reported to refine the electronic structure and catalytic behavior of Co─NC catalysts for high‐performance Zn–air batteries with impressive durability over 1700 h. Moreover, the optimized MgCo─NC cathode enables flexible Zn–air batteries with a mere 2% specific capacity degradation when the temperature fluctuates from 25 to −20 °C.

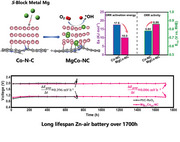

## 1 Introduction

The increasing environmental problems and energy crisis urgently require the development of efficient energy storage and conversion devices, such as metal‐air batteries and electrolyzers.^[^ ^1^, ^2^ ^]^ Among them, rechargeable Zn–air batteries (ZABs) have received widespread attention due to their formidable safety profile, affordability, benign environmental impact, and an impressive theoretical energy density that reaches a staggering 1086 Wh kg^−1^.^[^ ^3^, ^4^, ^5^ ^]^ Nevertheless, the path to integrating ZABs into our energy matrix is fraught with challenges. A particularly vexing obstacle is the suboptimal energy efficiency and narrow voltage windows, which result from the inherently slow kinetics of the cathodic oxygen reduction reaction (ORR).^[^ ^6^, ^7^, ^8^ ^]^ The Pt‐based catalysts have shown excellent electrocatalytic ORR activity, but their widespread commercial application is hampered by scarcity issues and a propensity for degradation under operational conditions.^[^ ^9^, ^10^ ^]^ Recently, single‐atom electrocatalyst and metal nanoparticles (NPs) coupled transition metal─N─C (TM─N─C, M = Fe, Co, Ni, Mn, Cu, etc.) materials have emerged as alternatives to Pt‐based catalysts due to their compositional and structural diversity, high activity, and impressive electrical conductivity.^[^ ^11^, ^12^, ^13^, ^14^ ^]^

Generally, the intrinsic ORR catalytic activity of TM─N─C catalysts is predominantly determined by the interactions between O‐intermediates (e.g. ^*^O_2_, ^*^OOH, ^*^OH, and ^*^O) and the active centers.^[^ ^15^, ^16^, ^17^ ^]^ The core of these interactions lies the coupling between the adsorptive characteristics of O 2*p* orbitals and the electronic states of TM *d* orbitals, which produce deep‐lying filled bonding states and partially occupied anti‐bonding states.^[^ ^18^ ^]^ The occupancy of anti‐bonding states governs the strength of these pivotal interactions, where an equilibrium neither too strong nor too weak is required for accelerating electrocatalytic processes.^[^ ^19^, ^20^ ^]^ Strategically, lowering the TM *d*‐band center to increase the occupancy anti‐bonding orbital is regarded as a compelling approach to weaken the overstrong interaction between TM sites and O‐intermediates, thereby augmenting its intrinsic catalytic activity.^[^ ^21^, ^22^, ^23^ ^]^ For instance, Li et al.^[^ ^24^ ^]^ demonstrated that the *d‐*band center of Co (−1.89 eV) in Cu/Co─N─C is more negatively shifted compared to that of Co (−1.83 eV) in Co─NC. This leads to an increased anti‐bonding orbital occupancy and reduced adsorption energy of O‐intermediates, thus boosting ORR activity. Complementary to these experimental findings, density functional theory (DFT) calculations by Li and co‐workers^[^ ^25^ ^]^ elucidated that *d*‐*d* orbital hybridization between Fe‐Mn diatomic pairs could refine the electronic structure of the active component, reduce the ^*^OH adsorption energy and expedite the ORR process. Additionally, substantial efforts have validated that *p*‐block elements such as Se,^[^ ^26^ ^]^ Sn,^[^ ^27^ ^]^ and Bi^[^ ^28^ ^]^ are capable of downshifting the *d*‐band center of TM sites through *p*‐*d* orbital hybridization thereby optimizing the energetics of the active center and oxygen intermediates. However, the inherent localized character of *p* and *d* orbitals results in an unsatisfactory modulation ability when hybridized with the active center's *d* orbitals. Recently, it was demonstrated that although *s*‐block metals are generally electrocatalytically inert, their delocalized *sp* orbitals offer opportunities in engineering M─N─C catalysts for improved activity and durability.^[^ ^29^, ^30^ ^]^ For instance, Tse and co‐workers reported an *s‐*group metal Mg‐doped Co─N─C for robust oxygen evolution.^[^ ^31^ ^]^ Hou and co‐workers reveal that Mg modulates the electron spin of the Fe site in Fe─N─C to enhance OER activity for overall water splitting.^[^ ^32^ ^]^ Nevertheless, an in‐depth investigation of the synergistic effect of the *s‐*group metal with TM─N─C for boosting oxygen reduction reaction (ORR), which is crucial for fuel cells and metal‐air batteries, is rarely realized to the best of our knowledge.

**Figure 1:**
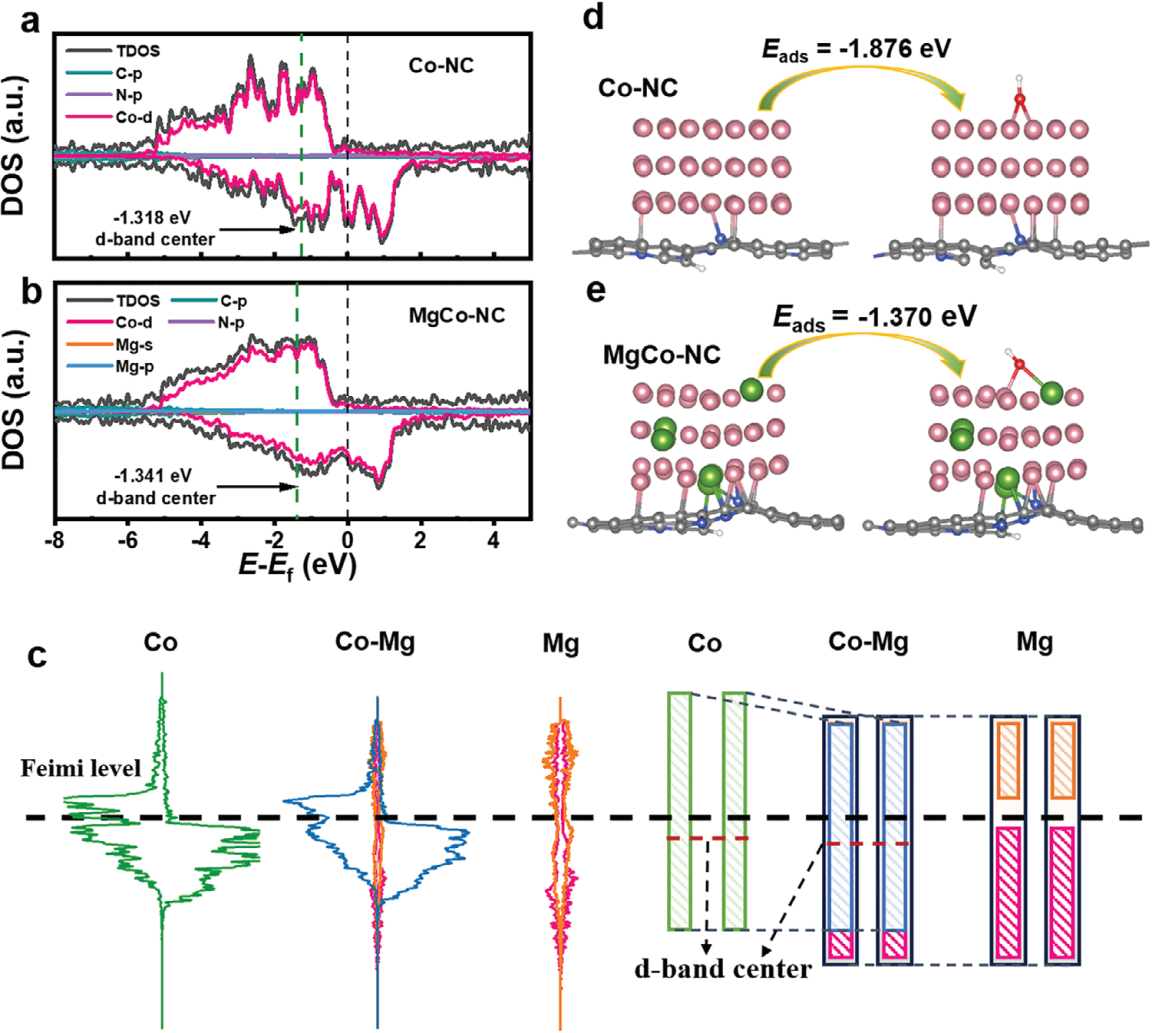
Density of states (DOS) of a) Co─NC and b) MgCo─NC. c) Orbital hybridization analysis. Calculated adsorption energy of ^*^OH on d) Co─NC and e) MgCo─NC. Color code: Mg (green), Co (pink), C (grey), N (blue), O (red) and H (white).

With these in mind, the effect of *s‐*block metal Mg on the electronic structure of Co in Co nanoparticle‐Co─N_4_─C composite (Co─N─C) was first explored. Here we choose Mg owing to its essential cofactor in biological enzymes, which has proven to have a suitable affinity for oxygenated species.^[^ ^33^ ^]^ The density of states (DOS) of Co─NC and MgCo─NC (**Figure** **1**a,b) reveals that the *d*‐band center of Co in MgCo─NC is positioned at a lower energy (−1.341 eV) compared to Co─NC (−1.318 eV). This shift potentially increases antibonding orbital occupancy and weakens the over‐strong interaction between the Co center with the oxygen intermediates. An in‐depth analysis of the DOS of the samples (Figure 1c) indicates that the observed lowering of the *d*‐band center of Co in MgCo─NC is caused by the hybridization of Mg *sp* orbitals and Co 3*d* orbitals.^[^ ^34^ ^]^ The binding energy of intermediate, specifically ^*^OH, at the active site is generally recognized as a critical determinant of ORR efficiency.^[^ ^35^, ^36^, ^37^ ^]^ Figure 1d,e showcases the adsorption energy of ^*^OH on the Co and Mg‐Co sites, respectively. Notably, the incorporation of Mg in Co─NC reduces the ^*^OH adsorption energy from −1.876 to −1.370 eV, thereby contributing to the promotion of the ORR process.^[^ ^38^, ^39^ ^]^

Herein, we report the incorporation of *s*‐block Mg into the CoN─C backbone (MgCo─NC) to afford remarkable ORR activity and stability. Impressively, the Zn–air battery based on MgCo─NC air electrode demonstrates a high peak power density of 157.0 mW cm^−2^ and an exceptional durability of 1700 h. Further optimized Mg_0.1_Co_0.9_─NC possesses a low ORR activation energy (*E*_a_ = 10.0 kJ mol^−1^), allowing satisfactory Zn–air battery power density and capacity at even −20 °C, outperforming the benchmark Pt/C+RuO_2_ couple, showcasing promising potential for practical applications in complex climate conditions.

## 2 Results and Discussion

### 2.1 Morphological and Structural Characterization

**Figure 2:**
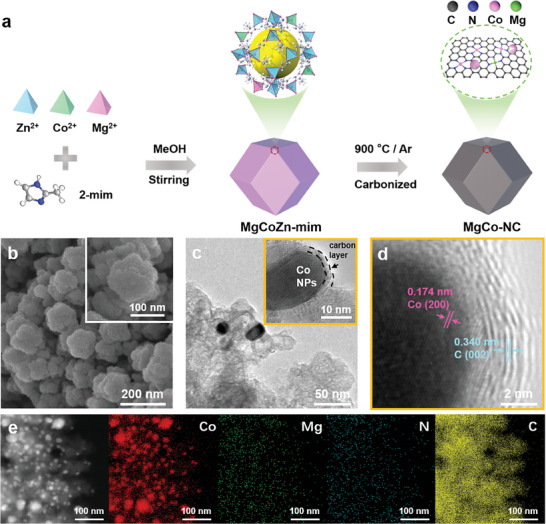
a) Schematic illustration for the preparation of MgCo─NC catalysts. b) SEM, c) TEM and d) HRTEM image of Mg_0.1_Co_0.9_─NC. e) EDS elemental mappings of Mg_0.1_Co_0.9_─NC.

The synthetic route of the MgCo─NC catalysts is illustrated in **Figure** **2**a. First, MgCoZn‐dimethylimidazole (MgCoZn‐mim) is synthesized by the reaction of mixed divalent metal cations (Mg^2+^, Co^2+,^ and Zn^2+^) and dimethylimidazole at room temperature. Specifically, Mg^2+^: Co^2+^: Zn^2+^ in molar ratios of 0.1: 0.9: 9, 0.5: 0.5: 9 and 0: 1: 9 affords Mg_0.1_Co_0.9_Zn_9_‐mim, Mg_0.5_Co_0.5_Zn_9_‐mim, and Co_1_Zn_9_‐mim, respectively. X‐ray diffraction (XRD) patterns in Figure S1 (Supporting Information) reveal that both the synthesized Mg_0.1_Co_0.9_Zn_9_‐mim and Mg_0.5_Co_0.5_Zn_9_‐mim share a similar crystalline structure with Co_1_Zn_9_‐mim. Subsequently, the precursors were calcined at 900 °C in an N_2_ atmosphere to obtain Mg_0.1_Co_0.9_─NC, Mg_0.5_Co_0.5_─NC, and Co─NC catalysts, respectively.

The morphologies of the MgCoZn‐mim precursors and the MgCo─NC catalysts are characterized by scanning electron microscopy (SEM) (Figures S2 and S3, Supporting Information). The precursors show a smooth polyhedral structure, while the annealed catalysts display a morphology similar to that of MgCoZn‐mim precursors (Figure 2b). The transmission electron microscopic (TEM) analysis (Figure 2c) reveals Co nanoparticles of ≈ 20 nm in diameter encapsulated in a carbon layer. In addition, the high‐resolution transmission electron microscopy (HRTEM) image of Mg_0.1_Co_0.9_─NC in Figure 2d shows two sets of lattice stripes at 0.174 nm corresponding to the Co (200) crystal plane and 0.340 nm corresponding to the C (002) crystal plane, respectively. The elemental distribution of Mg_0.1_Co_0.9_─NC is further investigated by TEM‐EDX elemental mapping images. As shown in Figure 2e, Co, Mg, N, and C elements are uniformly distributed in the Mg_0.1_Co_0.9_‐NC. Additionally, the molar ratio of Mg and Co in the Mg_0.1_Co_0.9_─NC is calculated as 1:7.25, according to inductively coupled plasma‐optical emission spectrometry (ICP‐OES) analysis.

**Figure 3:**
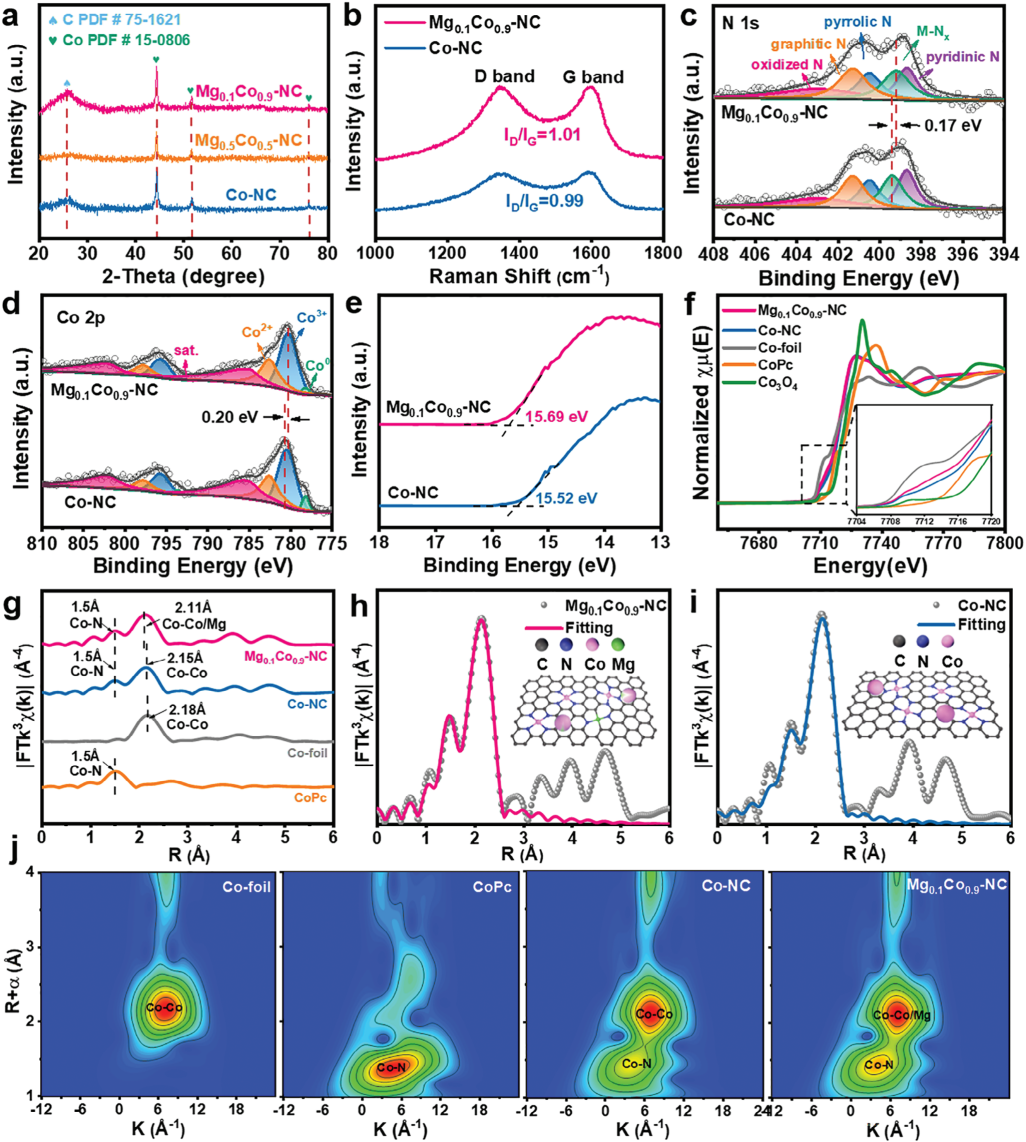
a) XRD pattern of Mg_0.1_Co_0.9_─NC, Mg_0.5_Co_0.5_─NC, and Co─NC. b) Raman spectra of Mg_0.1_Co_0.9_─NC and Co─NC. High‐resolution XPS spectras of c) N 1 s and d) Co 2p for Mg_0.1_Co_0.9_─NC. e) Ultraviolet photoelectron spectrometer (UPS) spectra of the catalysts. f) The Co K‐edge XANES spectra and g) the corresponding Fourier transform of k^3^‐weighted EXAFS spectra. h) Co‐EXAFS fitting results of Mg_0.1_Co_0.9_─NC at R space (inset, structural model of Mg_0.1_Co_0.9_─NC). i) Co‐EXAFS fitting results of Co─NC at R space (inset, structural model of Co─NC). j) Co K‐edge WT‐EXAFS spectra of Co foil, CoPc, Co─NC and Mg_0.1_Co_0.9_─NC.

The crystal structures of the MgCo─NC catalysts were characterized by powder X‐ray diffraction (XRD). As shown in **Figure** **3**a, the broad diffraction peak located at 2*θ* of 26.1° corresponds to the (002) plane of graphitic carbon (PDF#75‐1621),^[^ ^40^ ^]^ which originates from the carbonization of the 2‐methylimidazole ligand. The three diffraction peaks at 2*θ* = 44.3°, 51.9°, and 76.2° can be labeled as the (111), (200) and (220) planes of Co (PDF#15‐0806), respectively.^[^ ^41^, ^42^ ^]^ Figure 3b shows the Raman spectra of Mg_0.1_Co_0.9_─NC and Co─NC, where diffraction peaks at 1350 cm^−1^ and 1580 cm^−1^ correspond to the D and G peaks respectively. The I_D_/I_G_ of Mg_0.1_Co_0.9_‐NC is 1.01 higher than that of Co─NC (I_D_/I_G_ = 0.99), revealing that the Mg‐doped sample presents more defects, which is beneficial for enhancing the ORR activity of the electrocatalyst.^[^ ^43^, ^44^, ^45^ ^]^

To analyze the elemental composition and valence information of the samples, X‐ray photoelectron spectroscopy (XPS) characterizations of Mg_0.1_Co_0.9_─NC and Co─NC were performed. The XPS full spectrum (Figure S4, Supporting Information) demonstrates that the Mg_0.1_Co_0.9_─NC contains C, N, O, Co, and Mg elements. The deconvoluted N 1 s curves (Figure 3c) reveal five peaks of pyridine nitrogen (398.7 eV), metal─N (399.2 eV), pyrrole nitrogen (400.5 eV), graphite nitrogen (401.3 eV), and nitrogen oxide (403.1 eV).^[^ ^46^ ^]^ The M─N peaks of Mg_0.1_Co_0.9_─NC are shifted 0.17 eV to lower binding energy compared to Co─NC, implying that the introduction of Mg can modulate the electronic configuration of the M─N species, which may contribute to the ORR reaction.^[^ ^47^, ^48^ ^]^ The Mg 1 s spectrum (Figure S5, Supporting Information) of Mg_0.1_Co_0.9_─NC reveals that Mg exists in +2 valence, which can be deconvoluted into Mg‐C (1302.2 eV) and Mg‐N (1304.1 eV) peaks.^[^ ^49^ ^]^ The Co 2p_3/2_ high‐resolution spectrum (Figure 3d) shows four peaks at binding energies of 778.3, 780.3, 782.7, and 785.6 eV corresponding to Co^0^, Co^3+^, Co^2+^, and satellite peaks respectively.^[^ ^50^ ^]^ Compared to the Co─NC, the Mg_0.1_Co_0.9_─NC has a lower Co^0^ content, an increased Co^3+^ content, and a 0.2 eV shift of Co^3+^ to the low binding energy, which indicates that the introduction of Mg influences the electronic structure of Co. Electron paramagnetic resonance (EPR) measurements were performed to further investigate the electronic structures of Mg_0.1_Co_0.9_─NC and Co─NC. As shown in Figure S6 (Supporting Information), both Mg_0.1_Co_0.9_─NC and Co─NC show a broad peak with a g value of 2.442, which is due to the strong ferromagnetic properties of the Co nanoparticles. This also indicates that both Mg_0.1_Co_0.9_─NC and Co─NC have zero‐valent Co nanoparticles.^[^ ^50^ ^]^ This result is consistent with the Co 2p XPS result in Figure 3d. Ultraviolet photoelectron spectrometer (UPS) spectra (Figure 3e) display that the work function of Mg_0.1_Co_0.9_─NC is 5.53 eV, lower than that of Co─NC (5.7 eV). The reduced work function can be ascribed to the synergistic effect of Mg and Co─NC species, which may reduce the electron transfer energy barrier between oxygen intermediates and the catalysts.^[^ ^46^, ^51^ ^]^

The detailed electronic states and coordination environment of Co sites in Mg_0.1_Co_0.9_─NC and Co─NC were further probed by X‐ray absorption near‐edge structure (XANES) and extended X‐ray absorption fine structure (EXAFS) measurements, using Co‐foil, CoPc, and Co_3_O_4_ as reference samples. The XANES spectra (Figure 3f) reveal that the absorption profile of Mg_0.1_Co_0.9_─NC and Co─NC are located between Co‐foil (Co^0^) and Co_3_O_4_ (Co^2+^ and Co^3+^), indicating that the chemical valence state of Co is between 0 and +3. It is worth noting that compared to Co─NC, the profile of Mg_0.1_Co_0.9_─NC shifts toward lower energy, suggesting a lower oxidation state of Co element in Mg_0.1_Co_0.9_─NC. This result is consistent with the high‐resolution XPS spectra of Co 2p in Figure 3d. Fourier‐transformed k^3^‐weighted EXAFS (FT‐EXAFS) spectra of Mg_0.1_Co_0.9_─NC and Co─NC show an obvious peak at 1.5 Å (Figure 3g), ascribing to the Co─N coordination. In the higher R‐value region, the characteristic peak of Co nanoparticles appeared in both Mg_0.1_Co_0.9_─NC and Co─NC. The characteristic peak of Co‐metal in Mg_0.1_Co_0.9_─NC (2.11 Å) appears at a lower R‐value than that in Co─NC (2.15 Å), suggesting Mg is incorporated in Co nanoparticles. EXAFS fitting results display that the coordination number of Co─N in Mg_0.1_Co_0.9_─NC and Co─NC is ≈ 3.7 and 4.1, indicating the formation of the Co─N_4_ coordination structure (Figure 3h,i; Figures S7 and S8; Table S1, Supporting Information). Wavelet‐transformed EXAFS spectrum of Mg_0.1_Co_0.9_─NC shows obvious peaks at about 4.0 and 7.0 Å^−1^ in *k* space, further confirming the presence of Co─N and Co‐Co/Mg bonding (Figure 3j).

### 2.2 Electrocatalytic Performance of the Catalysts

**Figure 4:**
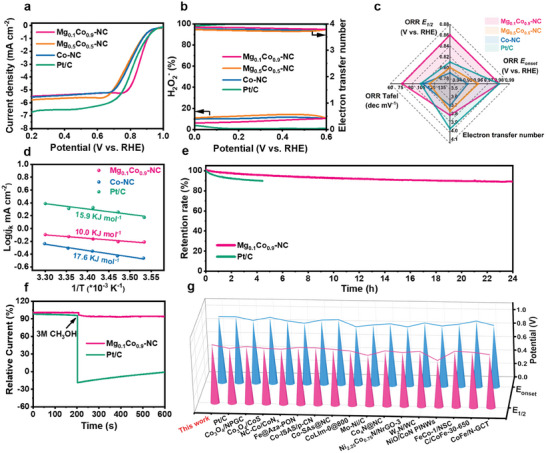
a) ORR polarization curves of Mg_0.1_Co_0.9_─NC, Mg_0.5_Co_0.5_─NC, Co─NC, and Pt/C. b) H_2_O_2_ selectivity and electron transfer numbers of the samples. c) Comparison of half‐wave potential, onset potential, electron transfer number, and Tafel slope of the catalysts. d) Arrhenius plots of Mg_0.1_Co_0.9_─NC, Co─NC and Pt/C. e) ORR stability test and f) methanol resistance tests of Mg_0.1_Co_0.9_─NC and Pt/C. g) Comparison of the ORR half‐wave potential and onset potential between Mg_0.1_Co_0.9_─NC and other recently reported electrocatalysts.

The ORR performances of the MgCo─NC catalysts were tested in a three‐electrode system with 0.1 m KOH alkaline electrolyte. Pt/C was used as a comparison sample to evaluate the performance of the MgCo─NC catalysts. As shown in Figure S9 (Supporting Information), MgCo─NC appears the redox peaks near 0.85 V vs. RHE in O_2_‐saturated electrolyte, whereas the peaks are absent in N_2_‐saturated electrolyte. The results demonstrate the potential redox performance of these catalysts. Linear scanning voltammetry (LSV) testing of the catalysts by rotating disc electrodes in **Figure** **4**a shows the outstanding ORR performance of Mg_0.1_Co_0.9_─NC (*E*_1/2_ = 0.86 V) compared to Mg_0.5_Co_0.5_─NC (*E*_1/2_ = 0.79 V), Co─NC (*E*_1/2_ = 0.80 V) and Pt/C (*E*_1/2_ = 0.81 V). The ORR selectivity of MgCo─NC catalysts was investigated by the rotating ring‐disk electrode (RRDE) test. The electron transfer number (Figure 4b) of the MgCo─NC catalysts are calculated to be 3.71∽3.99 in the range of 0∽0.6 V (vs. RHE), indicating that the MgCo─NC catalysts undergo four‐electron transfer ORR pathways. Figure S10 (Supporting Information) shows the LSV curves of Mg_0.1_Co_0.9_─NC at different rotational speeds. The number of transferred electrons can be calculated by the *K‐L* equation to be 3.95∽3.99,^[^ ^52^ ^]^ which also demonstrates the effective four‐electron ORR process of Mg_0.1_Co_0.9_─NC. The Mg_0.1_Co_0.9_─NC catalyst has a Tafel slope of 77.2 mV dec^−1^, lower than 117.1 mV dec^−1^ of Mg_0.5_Co_0.5_─NC, 107.5 mV dec^−1^ of Co─NC, and 104.2 mV dec^−1^ of Pt/C, indicating superior ORR kinetics of Mg_0.1_Co_0.9_─NC (Figure S11, Supporting Information). Accordingly, the introduction of an appropriate amount of Mg significantly enhanced the electrochemical performance of Co─NC, endowing Mg_0.1_Co_0.9_─NC with outstanding ORR activity, kinetics, and four‐electron selectivity (Figure 4c). Electrochemical impedance spectroscopy (EIS) is carried out to investigate the resistance of the electrodes. Nyquist plots in Figure S12 (Supporting Information) show that the Mg_0.1_Co_0.9_─NC possesses smaller charge transfer resistance (R_ct_, 36 Ω) than Mg_0.5_Co_0.5_─NC (39 Ω), Co─NC (44 Ω), and commercial Pt/C (83 Ω) in the high‐frequency region, indicating faster charge transfer ability and catalytic kinetics. To investigate the intrinsic activity of Mg_0.1_Co_0.9_─NC, the activation energies (*E*a) of the catalysts were assessed by the Arrhenius equation based on the temperature‐dependent (10∽30 °C) ORR polarization curves (Figure S13, Supporting Information). Specifically, *E*a of Mg_0.1_Co_0.9_─NC was calculated to be 10.0 kJ mol^−1^, which is much lower than 17.6 kJ mol^−1^ of Co─NC and 15.9 kJ mol^−1^ of Pt/C (Figure 4d) and other recent reports,^[^ ^46^, ^53^, ^54^, ^55^ ^]^ revealing the high intrinsic ORR activity of Mg_0.1_Co_0.9_─NC.

Stability is another significant factor in evaluating the performance of catalysts. The *i–t* curve (Figure 4e) at 0.6 V vs. RHE reveals that Mg_0.1_Co_0.9_─NC has a remarkable ORR stability with a current retention of 90% after 24 h of continuous testing. As a comparison, commercial Pt/C catalyst exhibits a current retention of 89% within 5 h of operation. Furthermore, Mg_0.1_Co_0.9_─NC has less fluctuation after adding 3 m CH_3_OH to the electrolyte at 200 s, whereas the current density of Pt/C dropped sharply in Figure 4f, proving the strong methanol resistance of Mg_0.1_Co_0.9_─NC. Notably, the ORR performance of Mg_0.1_Co_0.9_─NC is superior to many other recently reported non‐noble metal catalysts (Figure 4g; Table S2, Supporting Information).

### 2.3 Mg_0.1_Co_0.9_─NC‐Based Aqueous Zinc–air Batteries

**Figure 5:**
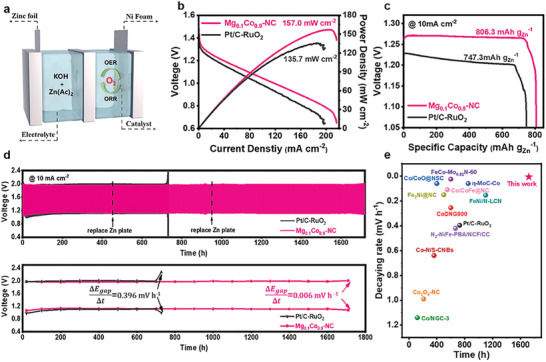
Performances of the ZABs using Mg_0.1_Co_0.9_─NC and Pt/C–RuO_2_ as air cathode. a) Scheme of the ZABs. b) Discharge polarization and power density curves. c) Curves of voltage versus specific capacity. d) Charge‐discharge cycling curves. e) Voltage variation versus cycling time. f) Comparison of the cycling stability and decaying rate of the Mg_0.1_Co_0.9_─NC‐based ZAB with other recent reports.

Inspired by the excellent ORR performance of Mg_0.1_Co_0.9_─NC, a rechargeable aqueous ZAB was assembled using the Mg_0.1_Co_0.9_─NC as an air cathode (**Figure** **5**a). A mixture of commercial catalysts Pt/C and RuO_2_ was used as the comparison sample. As shown in Figure S14 (Supporting Information), Mg_0.1_Co_0.9_─NC‐based ZAB exhibits a high open‐circuit voltage (1.46 V). Moreover, Mg_0.1_Co_0.9_─NC‐based ZAB shows a power density of up to 157.0 mW cm^−2^ at a current density of 206.3 mA cm^−2^ (Figure 5b), a specific capacity of 806.3 mAh g_Zn_^−1^ and an energy density of 1024 Wh kg_Zn_^−1^ at a discharge current of 10 mA cm^−2^ (Figure 5c), which is much better than those of Pt/C‐RuO_2_‐based ZAB (power density of 135.7 mW cm^−2^ and specific capacity of 747.3 mAh g_Zn_^−1^). As shown in Figure S15 (Supporting Information), the R_ct_ of Mg_0.1_Co_0.9_─NC‐based Zn–air battery is 38 Ω, lower than 80 Ω of the Pt/C‐RuO_2_‐based ZAB, suggesting higher charge‐transfer efficiency.

Figure S16 (Supporting Information) reveals the outstanding rate capability of Mg_0.1_Co_0.9_─NC‐based ZAB. It is worth mentioning that the aqueous Mg_0.1_Co_0.9_─NC‐based ZAB demonstrates a stable charge/discharge of 1700 h (5100 cycles, Figure 5d). In contrast, ZAB using commercial Pt/C‐RuO_2_ couple as a cathode can only operate stably for 720 h. Moreover, Mg_0.1_Co_0.9_─NC‐based ZAB achieved an ultra‐low voltage gap decaying rate of 0.006 mV h^−1^ at 10 mA cm^−2^ (Figure 5e), which is much lower than that of the Pt/C‐RuO_2_‐based ZAB (voltage gap decaying rate of 0.396 mV h^−1^). Impressively, as shown in Figure 5f and Table S3 (Supporting Information), the long cycling stability of the Mg_0.1_Co_0.9_─NC‐based ZAB is superior to many other recent reports.

### 2.4 Temperature Adaptability of Flexible ZABs

**Figure 6:**
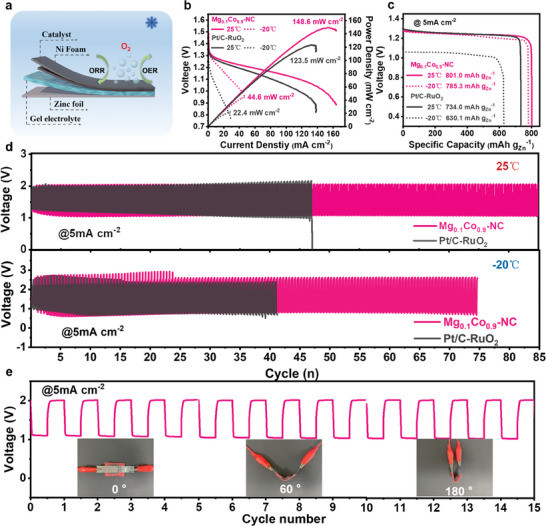
Performances of the flexible ZABs using Mg_0.1_Co_0.9_─NC and Pt/C‐RuO_2_ as air cathodes. a) Scheme of the flexible ZABs. b) Discharge polarization and power density curves. c) Specific capacity curves. d) Charge‐discharge cycling curves at 25 and −20 °C. e) Charge‐discharge cycling curves at different bending angles (0°, 60° and 180°).

To demonstrate the potential application of Mg_0.1_Co_0.9_─NC in wearable electronic devices, flexible ZABs were assembled using the catalyst as the cathode, acrylic acid (AA) hydrogel as the electrolyte, and the Zn plate as the anode (**Figure** **6**a). Mg_0.1_Co_0.9_─NC‐based ZAB exhibits a high open‐circuit voltage (1.45 V) in Figure S17 (Supporting Information). As shown in Figure 6b, Mg_0.1_Co_0.9_─NC‐based flexible ZAB demonstrates a high power density of 148.6 mW cm^−2^, exceeding 123.5 mW cm^−2^ of Pt/C‐RuO_2_‐based FZAB. At −20 °C, Mg_0.1_Co_0.9_─NC‐based flexible ZAB still has a higher power density (44.6 mW cm^−2^) than Pt/C‐RuO_2_‐based flexible ZAB. Impressively, the Mg_0.1_Co_0.9_─NC‐based flexible ZAB shows a high specific capacity of 801.0 mAh g_Zn_^−1^ at room temperature (25 °C) with a decay rate of only 2% (785.3 mAh g_Zn_^−1^) when the operation temperature is set at −20 °C (Figure 6c; Table S4, Supporting Information). It is worth noting that the Mg_0.1_Co_0.9_─NC‐based flexible ZAB can charge and discharge at −20 °C for a longer period than the control device based on Pt/C‐RuO_2_ (Figure 6d), demonstrating satisfactory temperature tolerance. Voltage fluctuations in the initial several cycles may be caused by insufficient electrode‐electrolyte interface compatibility in flexible ZAB.^[^ ^56^, ^57^ ^]^ Figure 6e shows that the flexible ZAB with Mg_0.1_Co_0.9_─NC cathode can be also operated under different bending degrees (0°, 60°, 180°). These results demonstrate the possibility of practical applications of high‐performance temperature‐tolerant Mg_0.1_Co_0.9_─NC‐based flexible ZABs in flexible electronics.

## 3 Conclusion

In summary, an innovative integration of *s*‐block element Mg into a cobalt‐nitrogen‐carbon (Co─N─C) framework was demonstrated, which proves a remarkable tunability in its electrocatalytic properties. DFT calculations revealed that the strategic *sp*‐*d* orbital hybridization between Mg and Co plays a pivotal role in the performance. This critical interaction effectively lowers the *d*‐band center of Co and weakens its adsorption energy with oxygen intermediates (^*^OH), further boosts the ORR activity. Through meticulous experimental optimization, the optimal molar ratio was achieved with an ideal composition of Mg_0.1_Co_0.9_─NC, which manifests a low apparent activation energy (10.0 kJ mol^−1^) and high ORR activity (half‐wave potential of 0.86 V). When applied as the cathode material of Zn–air battery, the Mg_0.1_Co_0.9_─NC‐based device delivers a high power density of 157.0 mW cm^−2^, and maintains a stable charge/discharge cycle over an extended duration of 1700 h at 10 mA cm^−2^. Particularly noteworthy is the Mg_0.1_Co_0.9_─NC‐based flexible ZAB showcasing outstanding properties and desirable low‐temperature adaptability. This work paves the way for the development of highly efficient, durable, and flexible energy devices capable of operating under a variety of environmental conditions.

## 4 Experimental Section

### Synthesis of Mg_0.1_Co_0.9_Zn_9_‐mim, Mg_0.5_Co_0.5_Zn_9_‐mim and Co_1_Zn_9_‐mim

In a typical synthesis, 0.0043 g Mg(CH_3_COO)_2_·4H_2_O, 0.0786 g Co(NO_3_)_2_·6H_2_O and 0.8032 g Zn(NO_3_)_2_·6H_2_O were dissolved in 30 mL of methanol, noted as liquid A, 1.968 g 2‐methylimidazole was dissolved in 30 mL of methanol, noted as liquid B. Liquid A and liquid B were mixed under vigorous stirring and stirred continuously for 8 h at room temperature (25 °C). Subsequently, the precipitate was obtained by washing with methanol and centrifuging three times, which was dried overnight in a vacuum oven at 60 °C to obtain Mg_0.1_Co_0.9_Zn_9_‐mim. The synthesis procedure of Mg_0.5_Co_0.5_Zn_9_‐mim and Co_1_Zn_9_‐mim is the same as that for Mg_0.1_Co_0.9_Zn_9_‐mim, except for the different dosing ratios of the metal salts.

### Synthesis of Mg_0.1_Co_0.9_─NC, Mg_0.5_Co_0.5_─NC, and Co─NC

Mg_0.1_Co_0.9_─NC, Mg_0.5_Co_0.5_─NC, and Co─NC were obtained by calcining Mg_0.1_Co_0.9_Zn_9_‐mim, Mg_0.5_Co_0.5_Zn_9_‐mim and Co_1_Zn_9_‐mim precursors under N_2_ atmosphere at 3 °C min^−1^ up to 900 °C for 2 h.

### Synthesis of Acrylic Acid (AA) Hydrogel

7.2 mL of acrylic acid (AA), 5 mL of 20 m NaOH, and 10 mL of deionized water were combined, then the mixture was sonicated for 30 min in the ice water bath to ensure thorough mixing. Subsequently, 0.11 g of K_2_S_2_O_8_ and 0.0077g N, N‐methylene bisacrylamide were added to the combined solution and sonicated for 30 min to dissolve it fully. The AA gel was produced by pouring the obtained solution into a glass mold to react for 20 min at 60 °C. The AA gel was immersed in 6 M KOH + 0.2 m Zn(Ac)_2_ for 3 days to obtain the gel electrolyte.^[^ ^58^ ^]^

### Material Characterization

The morphology of the samples was characterized by field emission scanning electron microscopy (FESEM, FEI Nova NanoSEM 450), transmission electron microscopy (TEM, JEM‐100CX II), and high‐resolution TEM (HRTEM, FEI Talos S‐FEG). The ratio of Mg and Co was detected by inductively coupled plasma‐optical emission spectroscopy (ICP‐OES, Agilent 720ES). The crystal structure of the catalysts was diffracted by powder X‐ray diffraction (XRD) patterns using a PANalytical X'Pert PRO with Cu K*α* incident radiation (*λ* = 0.1541 nm). The sample structure was analyzed using X‐ray photoelectron spectroscopy (XPS, Shimadzu Kratos Axis Ultra‐DLD), Raman spectra (Raman, LabRam HR Evolution), and electron paramagnetic resonance (EPR, Bruker EMX PLUS). Ultraviolet photoelectron spectra (UPS, E_ψ_ = 21.2 eV − E_cut‑off_) were recorded on a PHI5000 VersaProbe III (scanning ESCA microprobe) SCA (spherical analyzer).

### Electrocatalytic Characterization

All electrochemical performances were carried out by CHI 760E (CH Instruments, Inc., Shanghai, China) on a standard three‐electrode system with a counter electrode (carbon rod), a reference electrode (Ag/AgCl, 3 M KCl), and a working electrode (rotating disk electrode (RDE, 0.196 cm^2^) or rotating ring‐disk electrode (RRDE, 0.2475 cm^2^, N = 37%)). 2.5 mg of catalyst, 5 µL of Nafion (5.0 wt.%), 125 µL of deionized water, and 370 µL of ethanol were combined and sonicated for 40 min to create catalyst inks. The homogenized catalyst inks were added dropwise to RDE (8 µL) or RRDE (10 µL). The following formula is used to convert all potential values to potential versus RHE:

(1) $$E_{RHE}=E_{Ag/AgCl}+{E^{\theta }}_{Ag/AgCl}+0.059\times pH$$

Catalysts were tested in O_2_‐saturated and N_2_‐saturated 0.1 m KOH without rotational speed to obtain cyclic voltammetry (CV) curves. Linear sweep voltammetry (LSV) polarisation curves for different catalysts were obtained in O_2_‐saturated 0.1 m KOH at 1600 rpm with a scan rate of 5 mV s^−1^. The number of electron transfers (n) and the H_2_O_2_ selectivity (%) of the catalysts were measured by rotating the ring‐disk electrode. These values were computed using the following Equations (2) and (3):

(2) $$n\;=\frac{4\times I_{disk}}{\left(I_{disk}+\frac{I_{ring}}{N}\right)}$$

(3) $$H_{2}\;O_{2}=\frac{200\times \left(\frac{I_{ring}}{N}\right)}{\left(I_{disk}+\frac{I_{ring}}{N}\right)}$$

In addition, the electron transfer number (n) of the ORR can be obtained by the Koutecký–Levich (K–L) equation.^[^ ^40^ ^]^ Electrochemical impedance spectroscopy (EIS) was tested at half‐wave potentials with a frequency range of 0.1∽100,000 Hz. The activation energy was obtained by testing the LSV of the catalyst at different temperatures (10∽30 °C) and calculating with the Arrhenius Equation, which is as follows^[^ ^44^ ^]^:

(4) $$Arrhenius\;plots:\;\;\left(\frac{\partial \left(log\left(j_{k}\right)\right)}{\partial \left(\frac{1}{T}\right)}\right)=\frac{E_{a}}{2.3R}$$

(5) $$j_{k}=\frac{j_{l}\times j}{j_{l}-j}$$

*j* : measured current density.

*j_k_* : kinetic current density.

*j_l_* : limited current density.

*R* : universal gas constant (8.314 J mol^−1^ K^−1^).

*I–t* curves were carried out in O_2_‐saturated 0.1 m KOH at 0.6 V vs RHE to assess catalyst stability. Similarly, *i–t* chronoamperometric analysis was performed by injecting 3 m methanol into the electrolyte at 200 s to test methanol resistance.

### Aqueous Zn–Air Battery Assembly

The aqueous Zn–air battery was assembled using a polished zinc plate (200 µm) as the anode, the solution of 6 m KOH + 0.2 m Zn(Ac)_2_ as the electrolyte and 1 cm^2^ carbon paper coated with 200 µL of catalyst ink (5 mg of catalyst, 40 µL of Nafion, 200 µL of ethanol, 760 µL of deionized water) as the cathode, fixed by a plastic mould.

### Flexible Zn–Air Battery Assembly

The flexible Zn–air battery was assembled using a polished zinc plate (80 µm) as the anode, the quasi‐solid electrolyte formed by AA gel immersed in 6 m KOH + 0.2 m Zn(Ac)_2_ solution and 1 cm^2^ carbon paper coated with 200 µL of catalyst ink (5 mg of catalyst, 40 µL of Nafion, 200 µL of ethanol, 760 µL of deionized water) as the cathode, fixed by 3M tape.

### Zn–Air Battery Performance Tests

The discharge polarization curves of Zn–air batteries were tested at a CHI 760E electrochemical workstation. The specific capacities and cycling stabilities of ZABs (current density of 10 mA cm^−2^, charge for 10 min and discharge for 10 min) and FZABs (current density 5 mA cm^−2^, charge for 10 min and discharge for 10 min) were tested on the Neware battery test station system.

### DFT Calculation

First‐principles^[^ ^59^, ^60^ ^]^ and spin‐polarization density functional theory (DFT) calculations were conducted using the generalized gradient approximation (GGA) with the Perdew‐Burke‐Ernzerhof (PBE).^[^ ^61^ ^]^ The Projected‐augmented‐wave (PAW) potentials^[^ ^62^, ^63^ ^]^ were used to represent ionic cores, and valence electrons were considered using a plane‐wave basis set with a kinetic energy cutoff of 450 eV.^[^ ^64^ ^]^ Partial occupancies of the Kohn‐Sham orbitals were permitted using the Gaussian smearing method with a width of 0.05 eV. Energy convergence threshold of 10^−5^ eV and force convergence threshold of 0.02 eV Å^−1^. The electronic energy was considered self‐consistent when the energy change was smaller than 10^−5^ eV, and the geometry optimization was considered convergent when the energy change was smaller than 0.02 eV Å^−1^. An 18 Å vacuum layer was added to the surface to eliminate artificial interactions between periodic images. The weak interaction was described by the DFT+D3 method, which incorporates empirical correction in Grimme's scheme.^[^ ^65^, ^66^ ^]^

## Conflict of Interest

The authors declare no conflict of interest.

## Supporting information

Supporting Information

## Data Availability

The data that support the findings of this study are available from the corresponding author upon reasonable request.
