# Supplementary material for: S‐Block Metal Mg‐Mediated Co─N─C as Efficient Oxygen Electrocatalyst for Durable and Temperature‐Adapted Zn–Air Batteries
Source: Adv Sci (Weinh). 2024 Jul 4;11(34):2403865. doi: 10.1002/advs.202403865 (PMC11425636; doi:10.1002/advs.202403865)
Supplement: Supplementary file 1 — Supporting Information [file ADVS-11-2403865-s001.docx]

**Supporting Information**

***S*-Block Metal Mg-Mediated Co**─**N**─**C as Efficient Oxygen Electrocatalyst for Durable and Temperature-Adapted Zn-Air Batteries**

Henan Wang, Xinxin Niu, Wenxian Liu,* Ruilian Yin, Jiale Dai, Wei Guo, Chao Kong, Lu Ma, Xia Ding, Fangfang Wu, Wenhui Shi, Tianqi Deng and Xiehong Cao,*

Henan Wang, Xinxin Niu, Wenxian Liu, Jiale Dai, Wei Guo, Chao Kong, Lu Ma, Xia Ding, Fangfang Wu, Xiehong Cao

College of Materials Science and Engineering, Pinghu Institute of Advanced Materials, Zhejiang University of Technology

Hangzhou 310014, P. R. China

E-mail: liuwx@zjut.edu.cn (Wenxian Liu); gcscaoxh@zjut.edu.cn (Xiehong Cao)

Ruilian Yin

College of Chemical Engineering, Zhejiang University of Technology

Hangzhou 310014, P. R. China

Wenhui Shi

Center for Membrane and Water Science and Technology, College of Chemical Engineering, Zhejiang University of Technology

Hangzhou 310014, P. R. China

Tianqi Deng

State Key Laboratory of Silicon and Advanced Semiconductor Materials & School of Materials Science and Engineering, Zhejiang University

Hangzhou 310027, P. R. China

Tianqi Deng

Institute of Advanced Semiconductors & Zhejiang Provincial Key Laboratory of Power Semiconductor Materials and Devices, Hangzhou Global Scientific and Technological Innovation Center, Zhejiang University

Hangzhou 311215, P. R. China


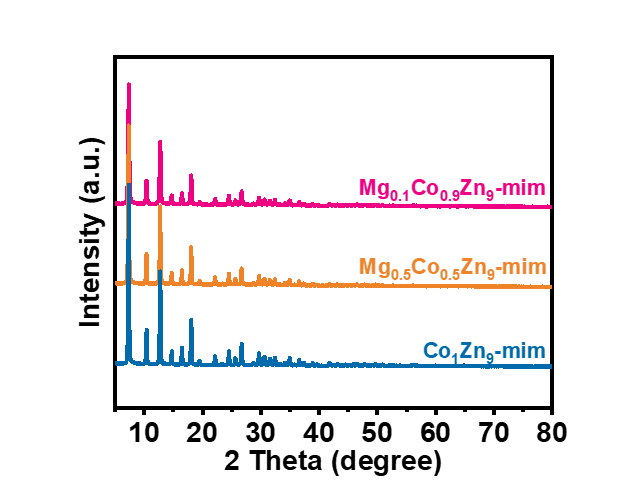


**Figure S1.** XRD pattern of Co_1_Zn_9_-mim, Mg_0.5_Co_0.5_Zn_9_-mim and Mg_0.1_Co_0.9_Zn_9_-mim.


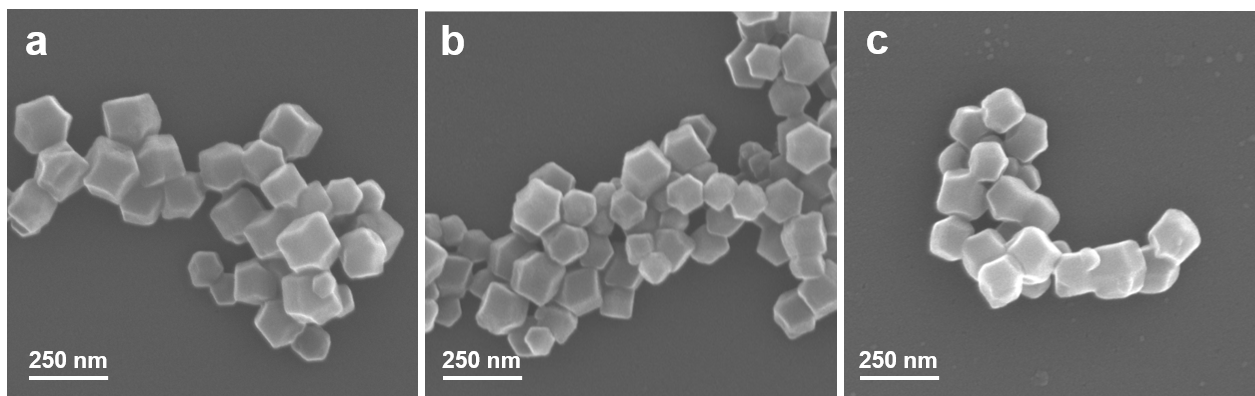


**Figure S2.** SEM images of a) Co_1_Zn_9_-mim, b) Mg_0.5_Co_0.5_Zn_9_-mim, c) Mg_0.1_Co_0.9_Zn_9_-mim.


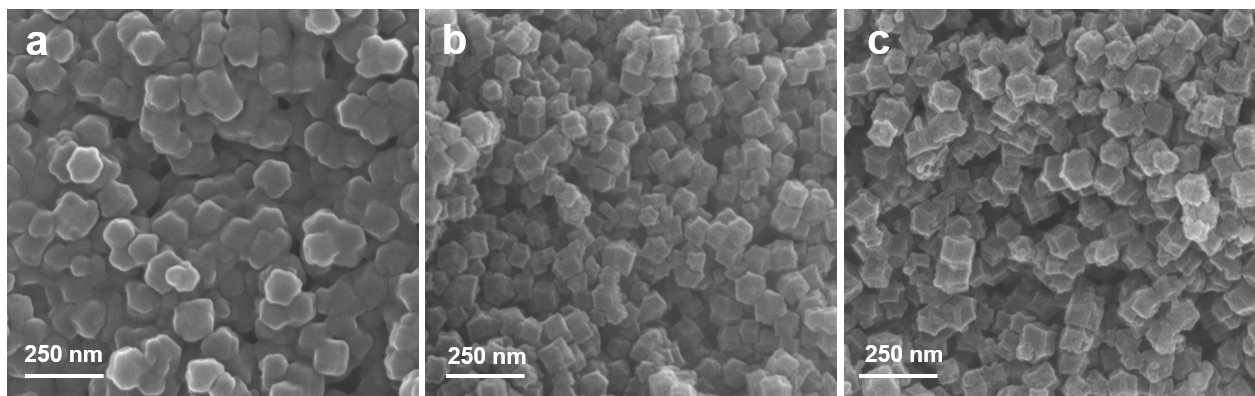


**Figure S3.** SEM images of a) Co─NC, b) Mg_0.5_Co_0.5_─NC and c) Mg_0.1_Co_0.9_─NC.


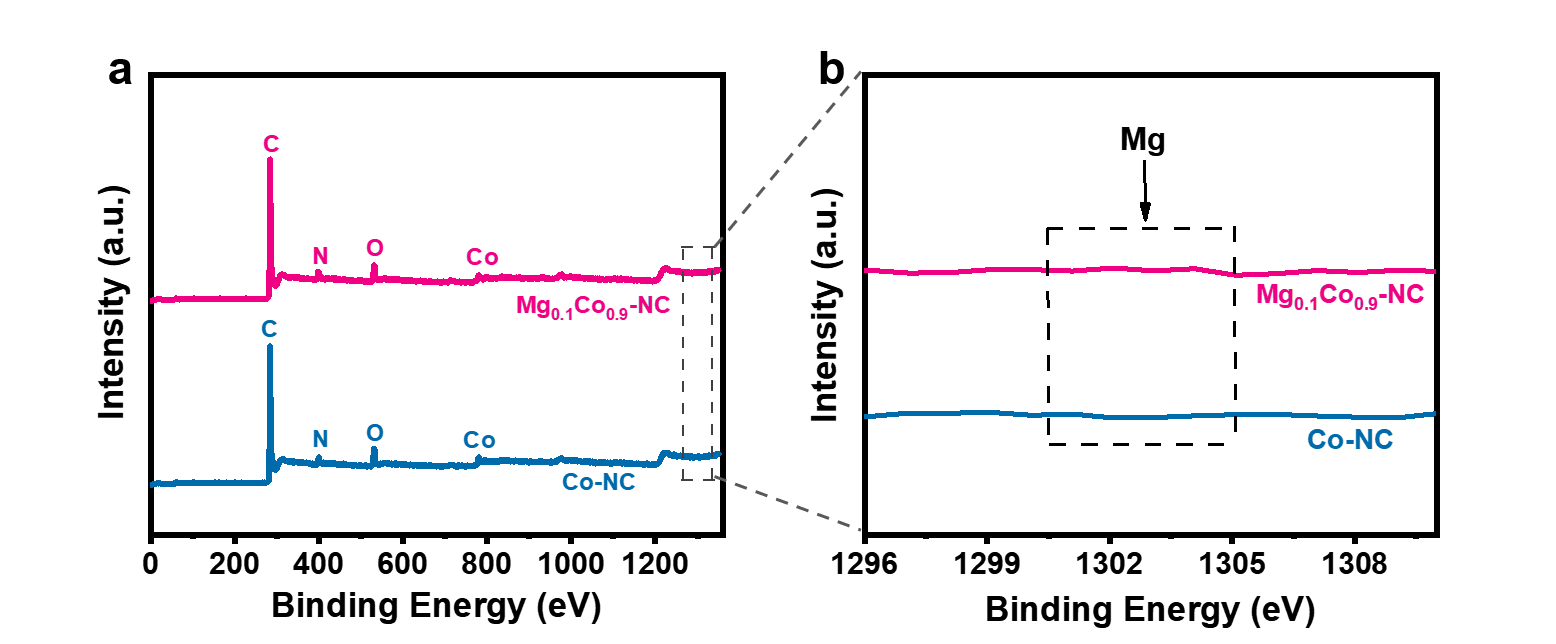


**Figure S4.** XPS survey spectra of Mg_0.1_Co_0.9_─NC and Co─NC catalysts.


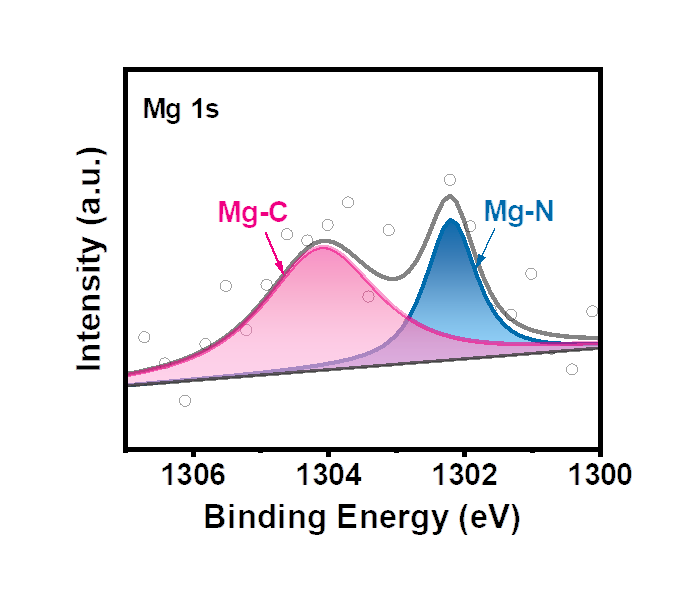


**Figure S5.** High-resolution XPS spectra of Mg 1s for Mg_0.1_Co_0.9_─NC.


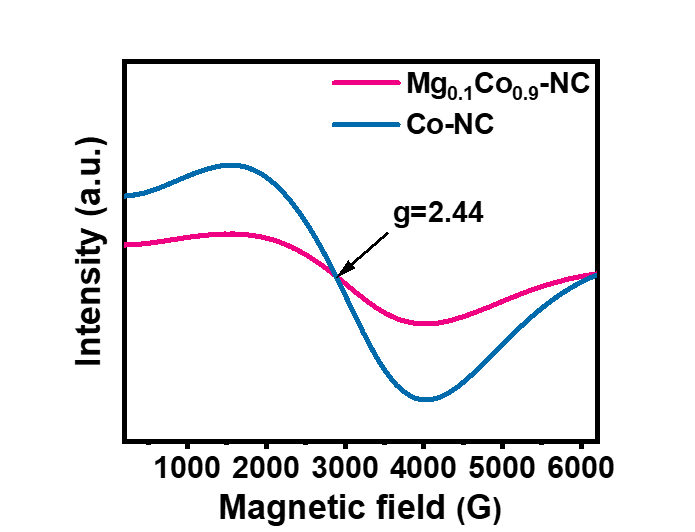


**Figure S6.** EPR spectra of Mg_0.1_Co_0.9_─NC and Co─NC


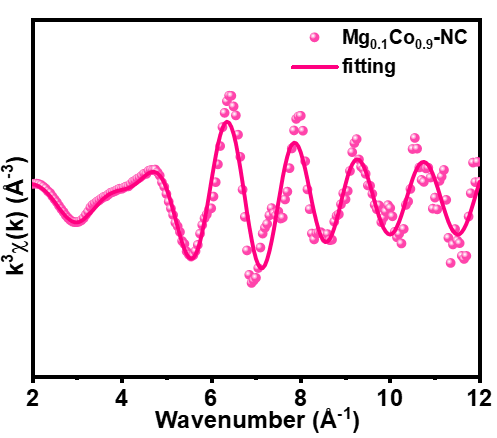


**Figure S7.** Co-EXAFS fitting curve of Mg_0.1_Co_0.9_─NC at K space.

EXAFS fitting was performed and analyzed with the Athena and Artemis programs of the Demeter data analysis packages [1] that utilize the FEFF6 program ^[2]^ to fit the EXAFS data. The energy calibration of the sample was conducted through standard and Co foil, which as a reference was simultaneously measured. A linear function was subtracted from the pre-edge region, then the edge jump was normalized using Athena software. The χ(k) data were isolated by subtracting a smooth, third-order polynomial approximating the absorption background of an isolated atom. The *k3*-weighted *χ(k)* data were Fourier transformed after applying a HanFeng window function (*Δk* = 1.0). For EXAFS modeling, the global amplitude EXAFS (*CN, R, σ*^2^ and Δ*E*_0_) was obtained by nonlinear fitting, with least-squares refinement, of the EXAFS equation to the Fourier-transformed data in *R*-space, using Artemis software, EXAFS of the Co foil was fitted. The obtained amplitude reduction factor *S_0_^2^* value (0.772) was set in the EXAFS analysis to determine the coordination numbers (*CNs*) in the Co-N, Co-Co, and Co-Mg scattering path in the sample.


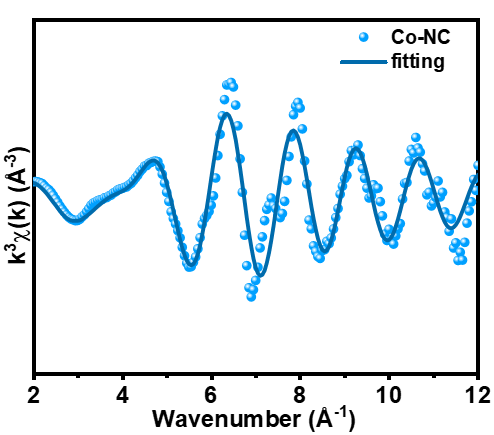


**Figure S8.** Co-EXAFS fitting curve of Co─NC at K space.


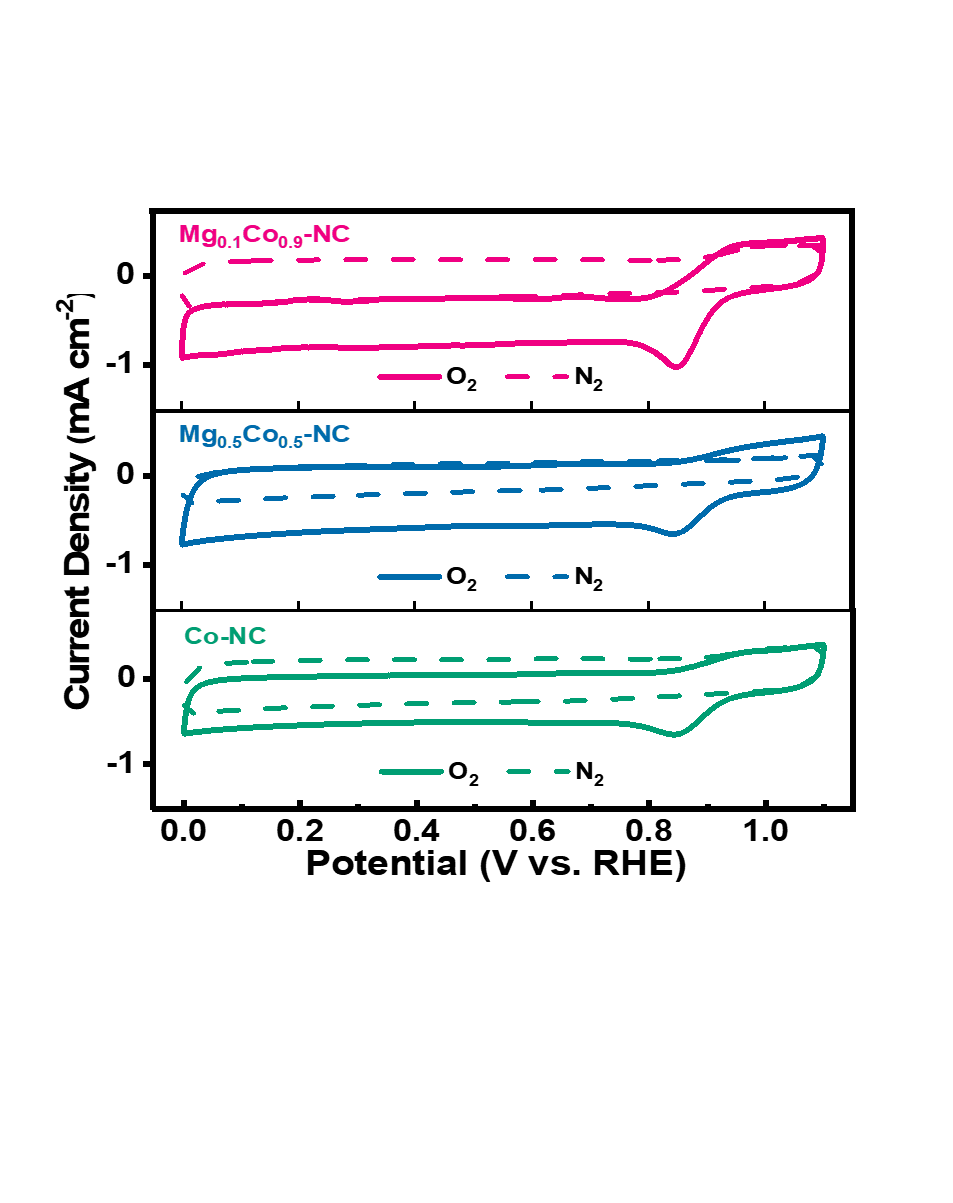


**Figure S9.** CV of Co─NC, Mg_0.5_Co_0.5_─NC and Mg_0.1_Co_0.9_─NC in O_2_ and N_2_-saturated 0.1 M KOH solution.


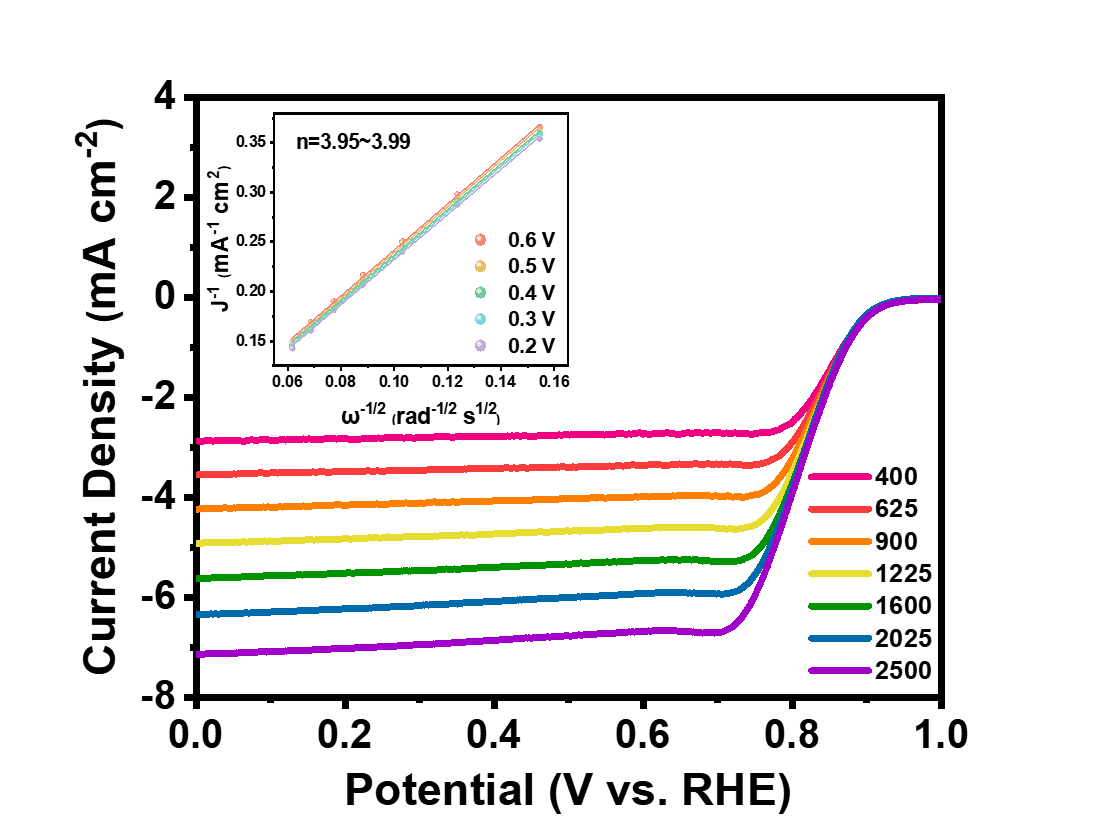


**Figure S10.** LSV curves of Mg_0.1_Co_0.9_─NC at different rotate speeds. Inset: the corresponding Koutecký-Levich plots.


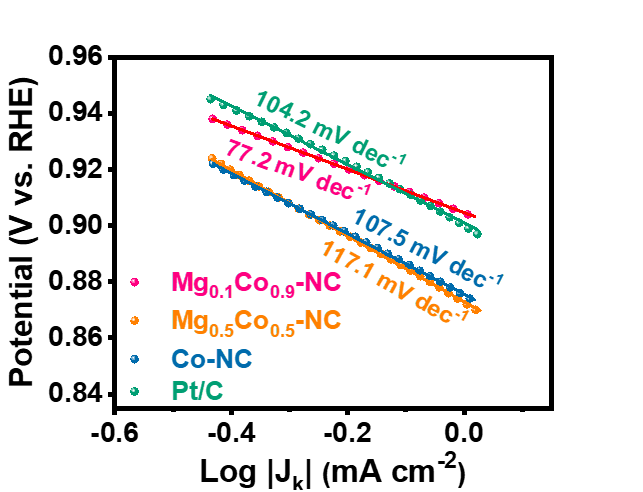


**Figure S11.** Tafel plots of Mg_0.1_Co_0.9_─NC, Mg_0.5_Co_0.5_─NC, Co─NC and Pt/C.


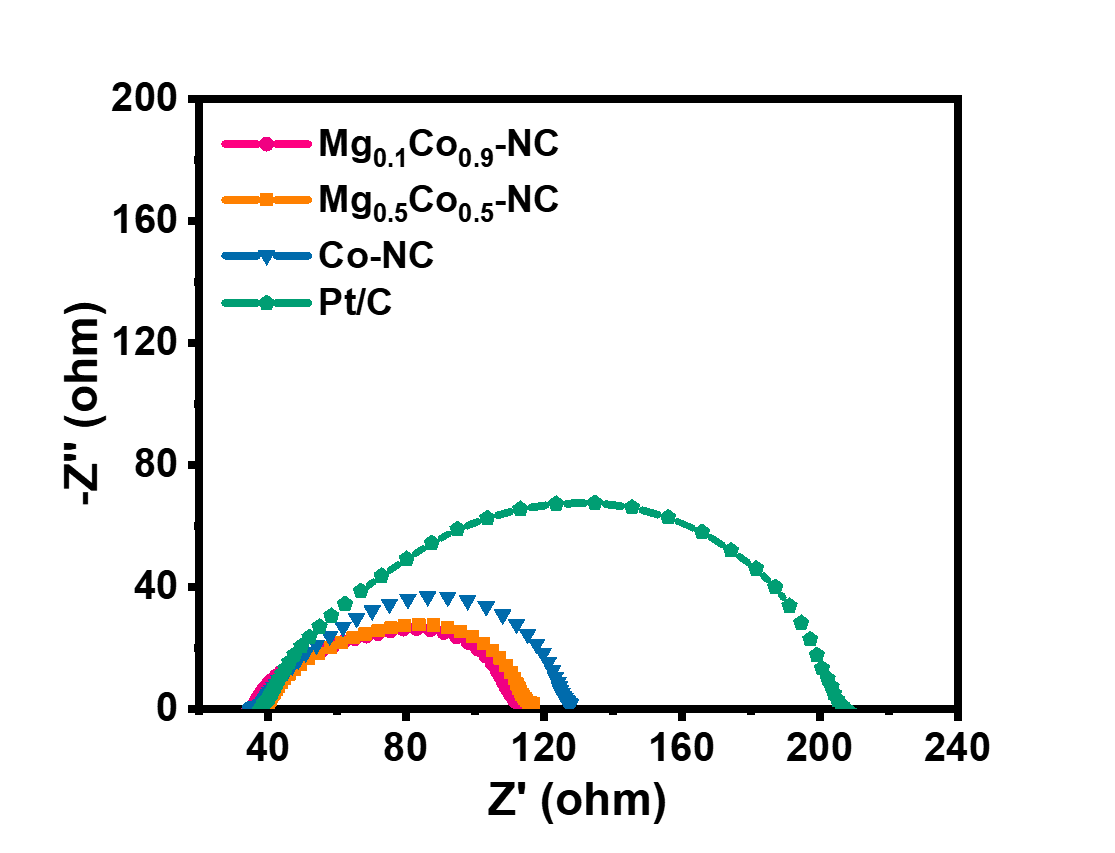


**Figure S12.** Electrochemical impedance spectroscopy (EIS) of MgCo─NC and Pt/C.


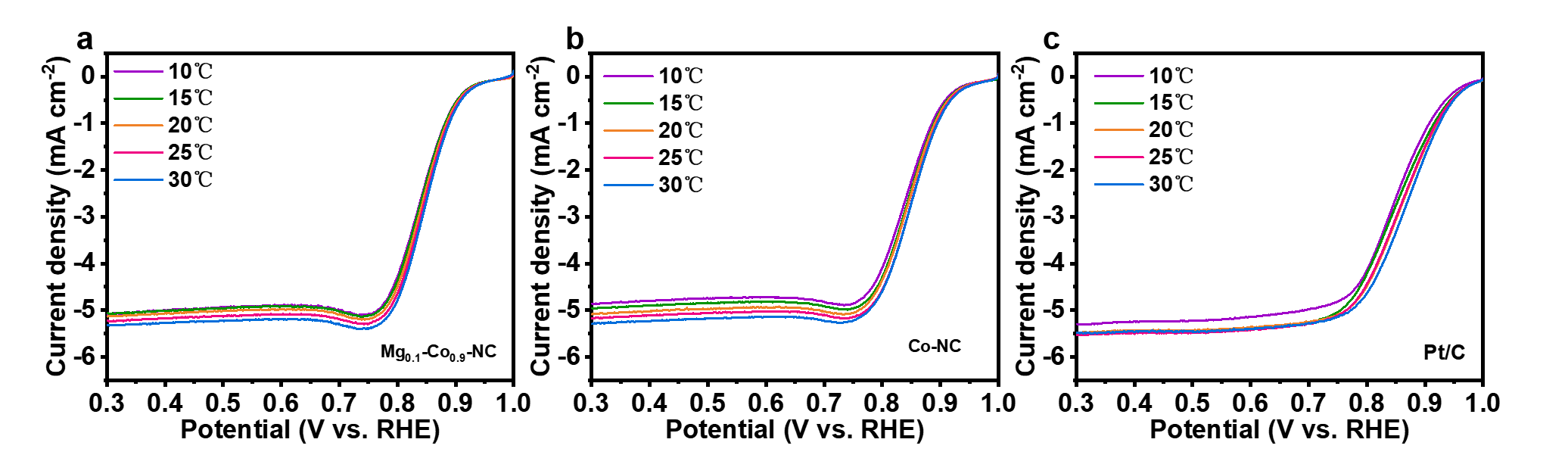


**Figure S13.** LSV of a) Mg_0.1_Co_0.9_─NC b) Co─NC and c) Pt/C at different temperatures (10°C~30°C).


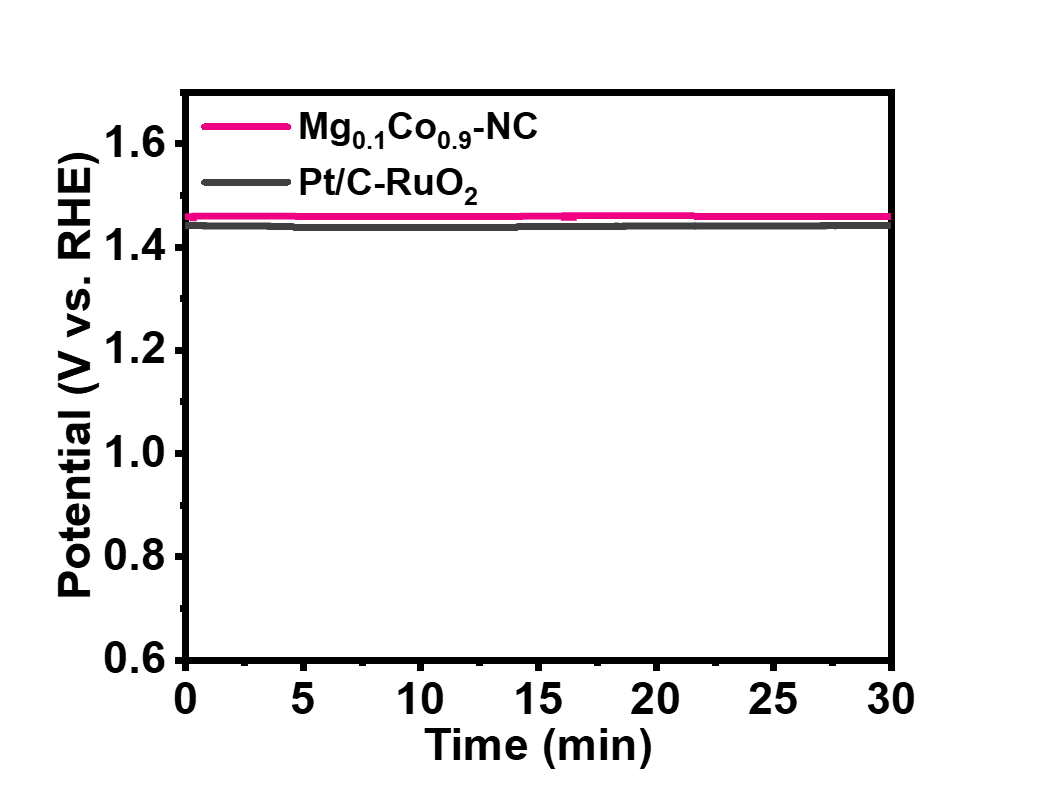


**Figure S14.** Open-circuit voltages of Mg_0.1_Co_0.9_─NC-based ZAB and Pt/C-RuO_2_-based ZAB.


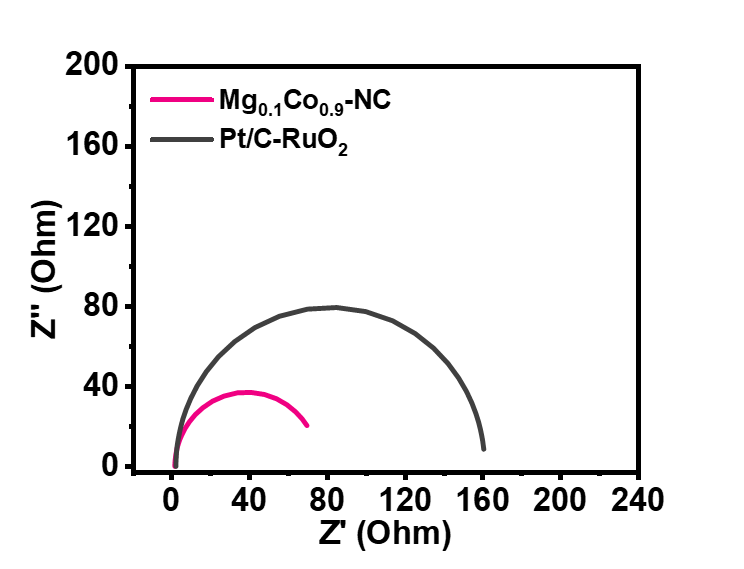


**Figure S15.** Electrochemical impedance spectroscopy (EIS) of the Zn-air batteries using Mg_0.1_Co_0.9_─NC and Pt/C-RuO_2_ as the cathode.


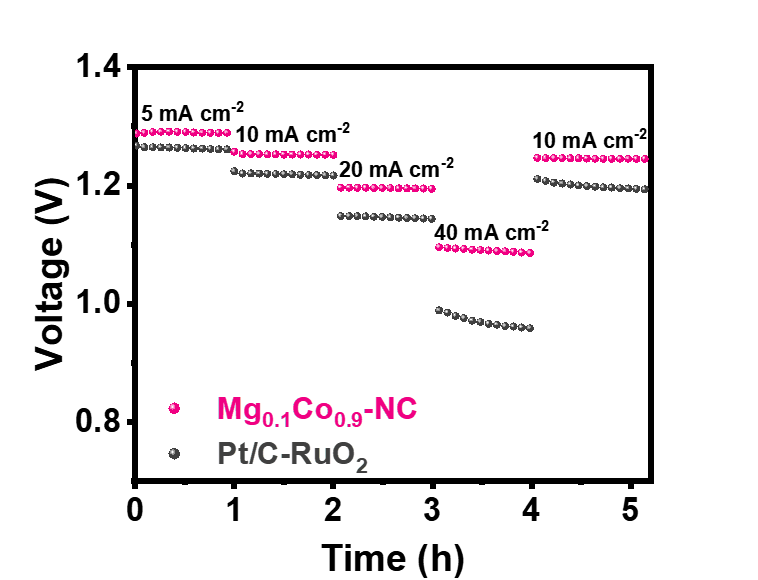


**Figure S16.** The rate performance of Mg_0.1_Co_0.9_─NC-based ZAB and Pt/C-RuO_2_-based ZAB.


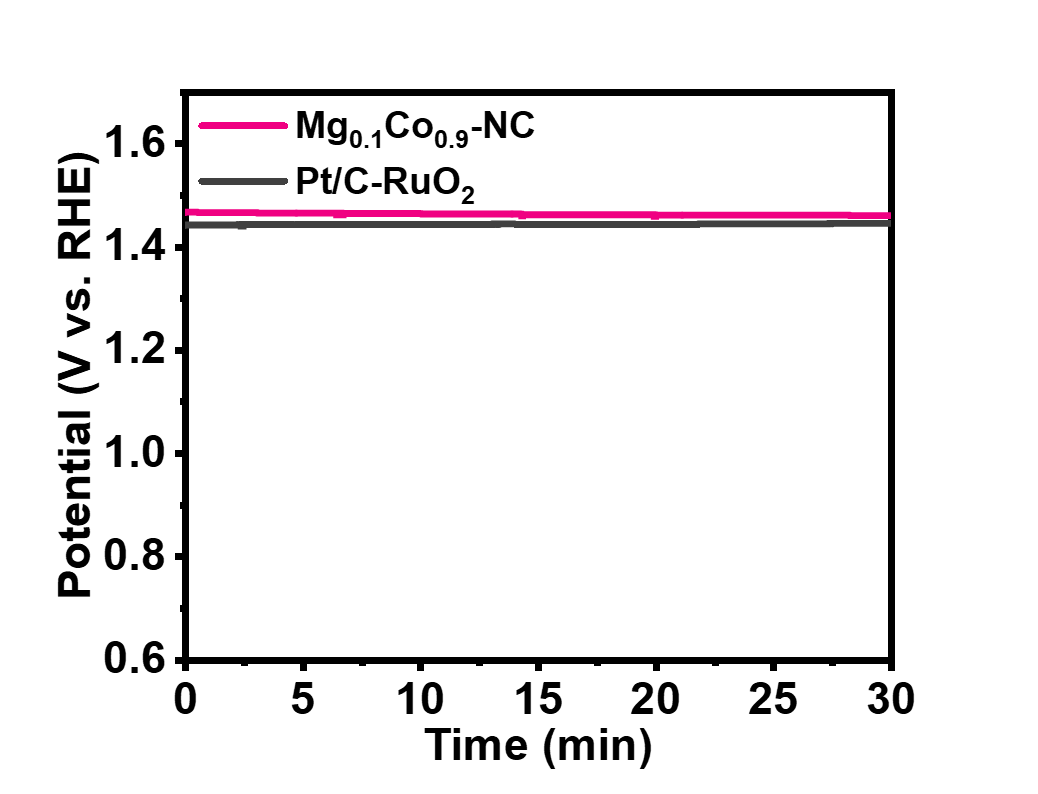


**Figure S17.** Open-circuit voltages of Mg_0.1_Co_0.9_─NC-based FZAB and Pt/C-RuO_2_-based FZAB.

**Table S1.** EXAFS fitting parameters at the Co K-edge of Mg_0.1_Co_0.9_─NC and Co─NC.

| Catalyst | Edge | Path | *CN* | *R*(Å) | *σ*^2^ (Å^2^) | *ΔE*_0_ (eV) | *R* factor |
| --- | --- | --- | --- | --- | --- | --- | --- |
| Mg_0.1_Co_0.9_─NC | Co | Co-N | 3.7 | 1.99 | 0.0096 | 1.2 | 0.0031 |
| Co─NC |  | Co-N | 4.1 | 2.00 | 0.0136 | -1.2 | 0.0077 |

*^a^CN*, coordination number; *^b^R*, the distance to the neighboring atom; *^c^σ*^2^, Debye-Waller factor, the Mean Square Relative Displacement (MSRD); *^d^ΔE*_0_, inner potential correction; *R* factor indicates the goodness of the fit. *S*_0_^2^ was fixed to 0.772, according to the experimental EXAFS fit of Co foil by fixing *CN* as the known crystallographic value.

**Table S2.** Comparison of ORR performance between Mg_0.1_Co_0.9_─NC and recent reports.

| Catalyst | E_1/2_  (V vs. RHE) | E_onset_  (V vs. RHE) | Reference |
| --- | --- | --- | --- |
| CoFe/N-GCT | 0.78 | 0.91 | ^[3]^ |
| C/CoFe-30-650 | 0.81 | 0.97 | ^[4]^ |
| FeCo-1/NSC | 0.82 | 0.92 | ^[5]^ |
| NiO/CoN PINWs | 0.68 | 0.89 | ^[6]^ |
| W_2_N/WC | 0.81 | 0.93 | ^[7]^ |
| Ni_2.25_Co_0.75_N/NrGO-3 | 0.79 | 0.87 | ^[8]^ |
| Co_4_N@NC | 0.81 | 0.91 | ^[9]^ |
| Mo-Ni/C | 0.74 | 0.89 | ^[10]^ |
| CoLIm-0@800 | 0.80 | 0.86 | ^[11]^ |
| Co-SAs@NC | 0.82 | 0.96 | ^[12]^ |
| Co-ISAS/p-CN | 0.838 | 0.95 | ^[13]^ |
| Fe@Aza-PON | 0.839 | 0.9 | ^[14]^ |
| NC-Co/CoNx | 0.97 | 0.93 | ^[15]^ |
| Co_3_O_4_/CoS | 0.82 | 0.978 | ^[16]^ |
| Co_3_O_4_/NPGC | 0.842 | 0.93 | ^[17]^ |
| Pt/C | 0.81 | 0.98 | This work |
| **Mg_0.1_Co_0.9_**─**NC** | **0.86** | **0.98** | **This work** |

**Table S3.** Comparison of Zn-air batteries stability between Mg_0.1_Co_0.9_─NC and recent reports.

| Catalyst | Cycle Time  (h) | Decay Rate  (mV h^-1^) | Reference |
| --- | --- | --- | --- |
| Co/CoO@NSC | 400 | 0.060 | ^[18]^ |
| GoDNG900 | 600 | 0.255 | ^[19]^ |
| η-MoC-900 | 850 | 0.060 | ^[20]^ |
| N_2_-NiFe-PBA/NCF/CC | 667 | 0.421 | ^[21]^ |
| Co/CoFe@NC | 550 | 0.109 | ^[22]^ |
| FeNi/N-LCN | 1100 | 0.154 | ^[23]^ |
| Co-N/S-CNBs | 360 | 0.640 | ^[24]^ |
| Fe_2_Ni@NC | 500 | 0.150 | ^[25]^ |
| Co_3_O_4_-NC | 210 | 0.990 | ^[26]^ |
| Co/NGC-3 | 120 | 1.140 | ^[27]^ |
| FeCo-Mo_0.82_N-60 | 600 | 0.025 | ^[28]^ |
| Pt/C-RuO_2_ | 720 | 0.396 | This work |
| **Mg_0.1_Co_0.9_**─**NC** | **1700** | **0.006** | **This work** |

**Table S4.** Comparison of flexible Zn-air batteries specific capacity between Mg_0.1_Co_0.9_─NC and recent reports.

| Catalyst | Specific Capacity  (mAh g_Zn_^−1^) | Temperature  (℃) | Reference |
| --- | --- | --- | --- |
| Pt/C-RuO_2_-PAM/PAA | 506 | -20 | ^[29]^ |
| FeCo-NPC | 687 | -20 | ^[30]^ |
| Fe_3_Co_7_-NC | 600 | -30 | ^[31]^ |
| Ni/N-ESC | 734 | -20 | ^[32]^ |
| PEMAC@NDCN | 765 | -20 | ^[33]^ |
| RuO_2_-Co_3_O_4_ | 506 | -20 | ^[34]^ |
| (Co,Fe)_3_O_4_ coated GDLs | 601 | -20 | ^[35]^ |
| Pt/C-RuO_2_ | 630 | -20 | This work |
| **Mg_0.1_Co_0.9_**─**NC** | **785** | **-20** | **This work** |

**References**

[1] B. Ravel, M. Newville, *J. Synchrot. Radiat.* **2005,** *12,* 537-541.

[2] Zabinsky, Rehr, Ankudinov, Albers, Eller, *Physical review. B, Condensed matter* **1995,** *52,* 2995-3009.

[3] X. Liu, L. Wang, P. Yu, C. G. Tian, F. F. Sun, J. Y. Ma, W. Li, H. G. Fu, *Angew. Chem. Int. Ed.* **2018,** *57,* 16166.

[4] F. Shi, K. Y. Zhu, X. K. Li, E. D. Wang, X. F. Zhu, W. S. Yang, *J. Energy Chem.* **2021,** *61,* 327.

[5] S. M. Chang, H. Zhang, Z. Y. Zhang, *J. Energy Chem.* **2021,** *56,* 64.

[6] J. Yin, Y. X. Li, F. Lv, Q. H. Fan, Y. Q. Zhao, Q. L. Zhang, W. Wang, F. Y. Cheng, P. X. Xi, S. J. Guo, *ACS Nano* **2017,** *11,* 2275.

[7] J. X. Diao, Y. Qiu, S. Q. Liu, W. T. Wang, K. Chen, H. L. Li, W. Y. Yuan, Y. T. Qu, X. H. Guo, *Adv. Mater.* **2020,** *32,* 1905679.

[8] Y. Q. He, X. H. Liu, A. L. Yan, H. Wan, G. Chen, J. L. Pan, N. Zhang, T. S. Qiu, R. Z. Ma, G. Z. Qiu, *ACS Sustain. Chem. Eng.* **2019,** *7,* 19612.

[9] L. L. Chen, Y. L. Zhang, X. J. Liu, L. Long, S. Y. Wang, X. L. Xu, M. C. Liu, W. X. Yang, J. B. Jia, *Carbon* **2019,** *151,* 10.

[10] J. Bejar, L. Alvarez-Contreras, M. Guerra-Balcazar, J. Ledesma-Garcia, L. G. Arriaga, N. Arjona, *Appl. Surf. Sci.* **2020,** *509,* 144898.

[11] A. Vilchez-Cozar, E. Armakola, M. Gjika, A. Visa, M. Bazaga-Garcia, P. Olivera-Pastor, D. Choquesillo-Lazarte, D. Marrero-Lopez, A. Cabeza, R. M. P. Colodrero, K. D. Demadis, *ACS Appl. Mater. Interfaces* **2022,** *14,* 11273.

[12] X. P. Han, X. F. Ling, Y. Wang, T. Y. Ma, C. Zhong, W. B. Hu, Y. D. Deng, *Angew. Chem. Int. Ed.* **2019,** *58,* 5359.

[13] A. J. Han, W. X. Chen, S. L. Zhang, M. L. Zhang, Y. H. Han, J. Zhang, S. F. Ji, L. R. Zheng, Y. Wang, L. Gu, C. Chen, Q. Peng, D. S. Wang, Y. D. Li, *Adv. Mater.* **2018,** *30,* 1706508.

[14] S. J. Kim, J. Mahmood, C. Kim, G. F. Han, S. W. Kim, S. M. Jung, G. M. Zhu, J. J. De Yoreo, G. Kim, J. B. Baek, *J. Am. Chem. Soc.* **2018,** *140,* 1737.

[15] C. Guan, A. Sumboja, W. J. Zang, Y. H. Qian, H. Zhang, X. M. Liu, Z. L. Liu, D. Zhao, S. J. Pennycook, J. Wang, *Energy Storage Mater.* **2019,** *16,* 243.

[16] K. Min, S. Kim, E. Lee, G. Yoo, H. C. Ham, S. E. Shim, D. Lim, S. H. Baeck, *J. Mater. Chem. A* **2021,** *9,* 17344.

[17] G. Li, X. L. Wang, J. Fu, J. D. Li, M. G. Park, Y. N. Zhang, G. Lui, Z. W. Chen, *Angew. Chem. Int. Ed.* **2016,** *55,* 4977.

[18] D. Zhou, H. Q. Fu, J. L. Long, K. Shen, X. L. Gou, *J. Energy Chem.* **2022,** *64,* 385.

[19] A. S. Wang, C. N. Zhao, M. Yu, W. C. Wang, *Appl. Catal. B-Environ.* **2021,** *281,* 119514.

[20] W. X. Liu, X. J. Dai, W. Guo, J. W. Tang, J. X. Feng, D. Zheng, R. L. Yin, Y. X. Wang, W. B. Que, F. F. Wu, W. H. Shi, X. H. Cao, *ACS Appl. Mater. Interfaces* **2023,** *15,* 41476.

[21] C. L. Lai, H. M. Li, Y. Sheng, M. Zhou, W. Wang, M. X. Gong, K. L. Wang, K. Jiang, *Adv. Sci.* **2022,** *9,* 2105925.

[22] Y. L. Niu, X. Teng, S. Q. Gong, M. Z. Xu, S. G. Sun, Z. F. Chen, *Nano-Micro Lett.* **2021,** *13,* 126.

[23] X. F. Li, Y. J. Liu, H. B. Chen, M. Yang, D. G. Yang, H. M. Li, Z. Q. Lin, *Nano Lett.* **2021,** *21,* 3098.

[24] M. W. Wang, L. Cao, X. Du, Y. Zhang, F. B. Jin, M. L. Zhang, Z. H. Li, K. M. Su, *ACS Appl. Mater. Interfaces* **2022,** *14,* 25427.

[25] J. B. Zhu, M. L. Xiao, G. R. Li, S. Li, J. Zhang, G. H. Liu, L. Ma, T. P. Wu, J. Lu, A. P. Yu, D. Su, H. L. Jin, S. Wang, Z. W. Chen, *Adv. Energy Mater.* **2020,** *10,* 1903003.

[26] C. Guan, A. Sumboja, H. J. Wu, W. N. Ren, X. M. Liu, H. Zhang, Z. L. Liu, C. W. Cheng, S. J. Pennycook, J. Wang, *Adv. Mater.* **2017,** *29,* 1704117.

[27] J. M. Li, Y. M. Kang, D. Liu, Z. Q. Lei, P. Liu, *ACS Appl. Mater. Interfaces* **2020,** *12,* 5717-5729.

[28] W. X. Liu, X. X. Niu, J. X. Feng, R. L. Yin, S. L. Ma, W. B. Que, J. L. Dai, J. W. Tang, F. F. Wu, W. H. Shi, X. J. Liu, X. H. Cao, *ACS Appl. Mater. Interfaces* **2023,** *15,* 15344.

[29] R. Chen, X. B. Xu, S. Y. Peng, J. M. Chen, D. F. Yu, C. H. Xiao, Y. L. Li, Y. T. Chen, X. F. Hu, M. J. Liu, H. Yang, I. Wyman, X. Wu, *ACS Sustain. Chem. Eng.* **2020,** *8,* 11501.

[30] Y. Zhou, Y. K. Liu, Z. L. Wang, C. Y. Li, Z. Y. Wang, S. Zhang, C. Deng, *Energy Storage Mater.* **2023,** *59,* 14.

[31] T. T. Gu, D. T. Zhang, Y. Yang, C. Peng, D. F. Xue, C. Y. Zhi, M. Zhu, J. Liu, *Adv. Funct. Mater.* **2023,** *33,* 11.

[32] Y. Zhou, M. X. Xie, Y. Song, D. K. Yan, Z. L. Wang, S. Zhang, C. Deng, *Energy Storage Mater.* **2022,** *47,* 235.

[33] K. Wagh Nayantara, S. Shinde Sambhaji, H. Lee Chi, H. Kim Sung, H. Kim Dong, D. Um Han, U. Lee Sang, H. Lee Jung, *Nano-Micro Lett.* **2022,** *14, 190*.

[34] D. Q. Jiang, H. Y. Wang, S. Wu, X. Y. Sun, J. Li, *Small Methods* **2022,** *6,* 2101043.

[35] J. Y. Cui, M. Labbe, H. J. Chung, D. G. Ivey, *J. Mater. Chem. A* **2023,** *11,* 13971.
